# Conjunctival epithelial cells resist productive SARS-CoV-2 infection

**DOI:** 10.1016/j.stemcr.2022.05.017

**Published:** 2022-06-23

**Authors:** Robert M. Jackson, Catherine F. Hatton, Jarmila Stremenova Spegarova, Maria Georgiou, Joseph Collin, Emily Stephenson, Bernard Verdon, Iram J. Haq, Rafiqul Hussain, Jonathan M. Coxhead, Hardeep-Singh Mudhar, Bart Wagner, Megan Hasoon, Tracey Davey, Paul Rooney, C.M. Anjam Khan, Chris Ward, Malcolm Brodlie, Muzlifah Haniffa, Sophie Hambleton, Lyle Armstrong, Francisco Figueiredo, Rachel Queen, Christopher J.A. Duncan, Majlinda Lako

**Affiliations:** 1 Biosciences Institute, Newcastle University, Newcastle Upon Tyne, UK; 2 Translational and Clinical Research Institute, Newcastle University, Newcastle Upon Tyne, UK; 3 National Specialist Ophthalmic Pathology Service (NSOPS) Department of Histopathology, E-Floor, Royal Hallamshire Hospital, Sheffield, UK; 4 Electron Microscopy Unit, Royal Hallamshire Hospital, Sheffield, UK; 5 NHS Blood and Transplant Tissue and Eye Services, Liverpool, UK; 6 Department of Dermatology and NIHR Newcastle Biomedical Research Centre, Newcastle Hospitals NHS Foundation Trust, Newcastle Upon Tyne, UK; 7 Wellcome Sanger Institute, Wellcome Genome Campus, Hinxton, Cambridge, UK; 8 Department of Ophthalmology, Royal Victoria Infirmary and Newcastle University, Newcastle, UK

**Keywords:** conjunctiva, conjunctival epithelium, SARS-CoV-2, ACE2, TMPRSS2, ocular surface, NFKB, IFN, productive infection

## Abstract

Conjunctival epithelial cells, which express viral-entry receptors angiotensin-converting enzyme 2 (ACE2) and transmembrane protease serine type 2 (TMPRSS2), constitute the largest exposed epithelium of the ocular surface tissue and may represent a relevant viral-entry route. To address this question, we generated an organotypic air-liquid-interface model of conjunctival epithelium, composed of basal, suprabasal, and superficial epithelial cells, and fibroblasts, which could be maintained successfully up to day 75 of differentiation. Using single-cell RNA sequencing (RNA-seq), with complementary imaging and virological assays, we observed that while all conjunctival cell types were permissive to severe acute respiratory syndrome coronavirus 2 (SARS-CoV-2) genome expression, a productive infection did not ensue. The early innate immune response to SARS-CoV-2 infection in conjunctival cells was characterised by a robust autocrine and paracrine NF-κB activity, without activation of antiviral interferon signalling. Collectively, these data enrich our understanding of SARS-CoV-2 infection at the human ocular surface, with potential implications for the design of preventive strategies and conjunctival transplantation.

## Highlights

• Human conjunctival epithelial cells are permissive to SARS-CoV-2 infection
• Human conjunctival epithelial cells resist productive SARS-CoV-2 infection
• Robust induction of NF-κB activity in SARS-CoV-2-infected and bystander conjunctival epithelium

## Graphical abstract

**Figure:**
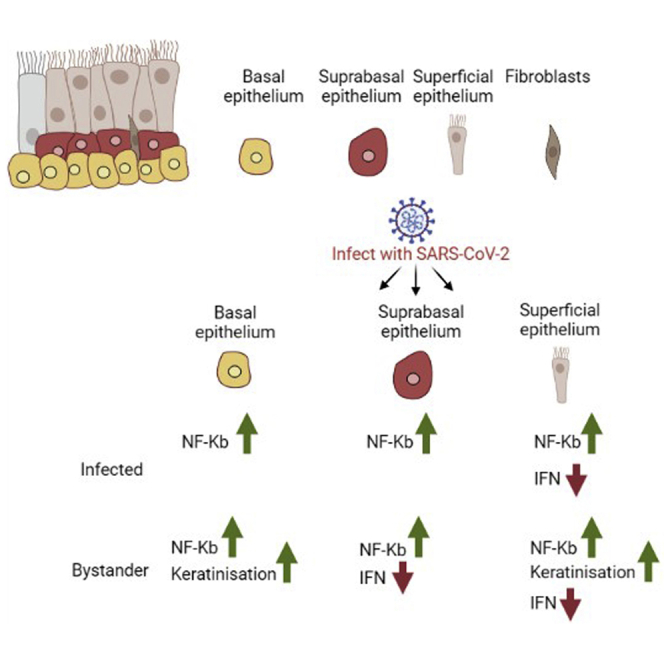

## Teaser

In this article, Lako and colleagues generated an air-liquid-interface *in vitro* model of human conjunctival cells comprising all the epithelial cell types found in the native conjunctiva. Infection of the model with SARS-CoV-2 shows that while all conjunctival cells can be infected with the virus, new viral particles that are necessary for propagation cannot be generated.

## Introduction

Coronavirus disease 2019 (COVID-19) is an infectious disease caused by the severe acute respiratory syndrome coronavirus 2 (SARS-CoV-2). It is well established that cells of the nasal and respiratory epithelium are the principal targets for SARS-CoV-2 (Sridhar and Nicholls, 2021). The ocular surface is a defined route of entry of several viral pathogens (Belser et al., 2013; Chu and Pavan-Langston, 1979). An important unresolved question is whether SARS-CoV-2 is similarly capable of infecting cells of the eye surface.

Host-cell-receptor expression is a major determinant of viral tropism. The SARS-CoV-2 spike protein binds angiotensin-converting enzyme 2 (ACE2), enabling viral entry, and spike-mediated membrane fusion is facilitated by the host transmembrane protease serine type 2 (TMPRSS2) (Hoffmann et al., 2020). Work from our group and others have demonstrated expression of key entry receptors by cells of the ocular surface (Collin et al., 2021b), suggesting that the eye may be a plausible route of viral entry. Evidence for ocular tropism of SARS-CoV-2 remains inconclusive, as recently reviewed (Armstrong et al., 2021). Clinical reports suggest that SARS-CoV-2 can be detected in tears and/or conjunctival swabs from patients with COVID-19, although the percentage of patients with detectable viral RNA was low (0%–5.3%) (Zhong et al., 2021; Seah et al., 2020). Clinical syndromes of ocular infection (e.g., conjunctivitis, keratoconjunctivitis, etc.) are also infrequently reported (Armstrong et al., 2021) in patient cohorts, although they are noted in case reports (Ozturker, 2021; Scalinci and Trovato Battagliola, 2020). Consistent with the relatively low frequency of detection of SARS-CoV-2 in clinical ocular specimens, a postmortem study identified SARS-CoV-2 RNA in ∼13% of 132 postmortem ocular tissues from 33 infected patients (Sawant et al., 2021). Conversely, in another postmortem study, viral protein was detected by immunofluorescence analysis in 3/3 patient ocular tissues analyzed, with positive staining found mainly in the limbus, and the central cornea exhibiting very low levels of viral detection (Eriksen et al., 2021). The ocular route appears to be a bona fide route of SARS-CoV-2 transmission in studies of rhesus macaques and Syrian golden hamsters (Imai et al., 2020; Deng et al., 2020; Hoagland et al., 2021). Ocular inoculation with SARS-CoV-2 resulted in a mild lung infection in these models; however, evidence for direct infection of the ocular surface was not sought (Deng et al., 2020). Notably, the nasolacrimal duct connects the ocular surface to the nasal mucosa, providing indirect access to nasal mucosal tissues from virus inoculated at the ocular surface; thus, ocular transmission does not necessarily imply permissiveness of the ocular surface.

Other studies have directly assessed the capacity of human ocular cells or tissues to support experimental infection *in vitro*. Miner and colleagues reported that human corneal cultures were resistant to SARS-CoV-2 infection (Miner et al., 2020). This resistance was not mediated by an innate antiviral type III interferon (IFN) response, as was the case for other viruses studied, suggesting alternate mechanism(s) of SARS-CoV-2 restriction. In compatible findings using cultured corneal, limbal, scleral, iris, retinal, and choroid cells from healthy cadaveric human donor eyes, alongside an induced pluripotent stem cell organoid system, Erikson and colleagues identified limbal cells to be more permissive than corneal cells to SARS-CoV-2 infection (Eriksen et al., 2021). Consistent with this, Sasamoto and colleagues demonstrated that limbal cells express high levels of ACE2 and TMPRSS2 (Sasamoto et al., 2021). The main limitation of the studies described above was that the conjunctiva, which occupies the largest ocular surface area and contains cells expressing *ACE2* and *TMPRSS2* (Collin et al., 2021b), was not investigated. To our knowledge, one recent study has assessed the permissiveness of conjunctival cells. In this study, Singh et al. dissected conjunctival cells and infected them under submerged culture conditions (Singh et al., 2021). They reported detection of viral RNA expression alongside expression of innate inflammatory mediators, suggestive of infection. They also detected spike protein in the superficial conjunctiva of patients that had succumbed to COVID-19. In cultures, the expression of viral protein declined rapidly from 24 to 72 h post-infection, suggesting that conjunctival cells may be unable to sustain infection. However, the capacity of these cells to support a productive infection, via assembly of nascent viral particles or release of infectious virus, and the responses of individual conjunctival cell types were not formally assessed. Further studies are required to determine whether the conjunctiva is a permissive tissue and might act as an entry portal for SARS-CoV-2.

Currently, there are limited *in vitro* cellular models available for modeling the human conjunctiva. Garíca-Posadas et al. developed two three-dimensional fibrin scaffolds on which they could seed human conjunctival epithelial cells (Garcia-Posadas et al., 2017). These models maintained their epithelial-like properties for 14 days before epithelial mesenchymal transition began, resulting in loss of MUC5AC expression by day 14. A slightly different approach was taken by Chung et al. to generate a multi-layered construct replicating the conjunctiva (Chung et al., 2007), comprised of a 6 to 8 layer epithelium with a high proportion of goblet cells. These constructs were characterized by the secretions of the membrane-bound MUC1, MUC4, and MUC16 and the secreted MUC5AC between 1 and 3 weeks of culture. The relative limitation was that replicative senescence was reached after 3 weeks and cells started to detach from the construct. An alternative model was developed to generate progenitor cells for use in transplantation. Conjunctival epithelial cells derived from patients were grown at the air-liquid interface (ALI), and after 2 weeks of differentiation expression of MUC5AC, KRT3, KRT19, and KRT12 could be detected by immunofluorescence analysis (Jeon et al., 2013). This tissue was used for transplantation in animal models, and consequently, longevity of the culture remains to be assessed.

To address definitively the permissiveness of conjunctival epithelium to SARS-CoV-2, we describe the generation and full characterization of an ALI organotypic conjunctival epithelial model composed of basal, suprabasal, and superficial epithelial cells, and fibroblasts. Using single-cell RNA sequencing (RNA-seq), with complementary imaging and virological assays, we define the cellular permissiveness of various epithelial cell types in the conjuctiva and determined the cell-type-specific innate immune response to SARS-CoV-2 infection.

## Results

### Generation and characterization of the ALI conjunctival epithelium model

**Figure 1:**
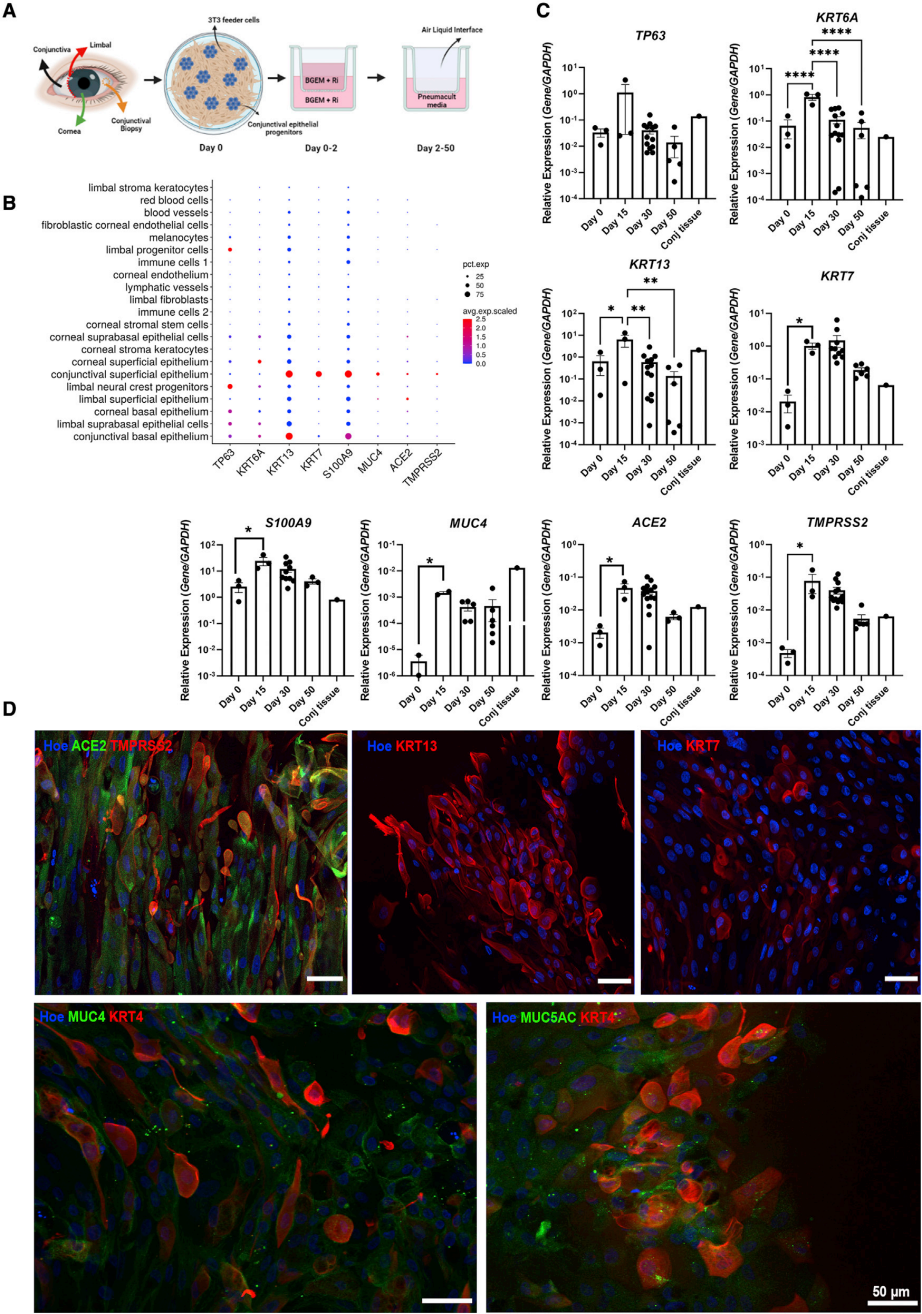
Generation and characterization of the ALI conjunctival organotypic culture model (A) Schematic summary showing the key steps involved in generation of the ALI conjunctival organotypic culture model. (B) RNA expression of conjunctival epithelial cell markers in the human adult cornea and conjunctiva single-cell RNA-seq data reported recently by Collin et al. (2021a). Raw expression values were normalized, log transformed, and summarized. The size of the dots indicates the proportion of cells, while the color indicates the mean expression. (C) Quantitative RT-PCR analysis showing expression of conjunctival cell-type-specific markers and SARS-CoV-2 entry factors (*ACE2* and *TMPRSS2*). Data shown as mean ± SEM, n = 3–14 experimental repeats in three different donors, ^∗^p < 0.05, ^∗∗^p < 0.01, ^∗∗∗^p < 0.001, one way ANOVA with Tukey’s multiple comparisons. Conj, conjunctiva. (D) Whole-mount immunofluorescence analysis showing expression of conjunctival epithelial marker (KRT13), superficial conjunctival epithelial marker (KRT4, KRT7), goblet cell marker (MUC5AC), mucin-producing cells (MUC4), and SARS-CoV-2 entry factors (ACE2, TMPRSS2) in day 15 ALI conjunctival organotypic culture models (representative of repeat experiments in three different donor conjunctival ALI cultures). Hoe, Hoescht. Scale bars 50 μm.

To develop a conjunctival organotypic model, epithelial cells from the perilimbal conjunctival epithelium were *ex vivo* expanded on mitotically inactivated 3T3 feeder cells (Spurr-Michaud and Gipson, 2013) (Figure 1A) and then matured at ALI conditions to induce differentiation.

**Figure 2:**
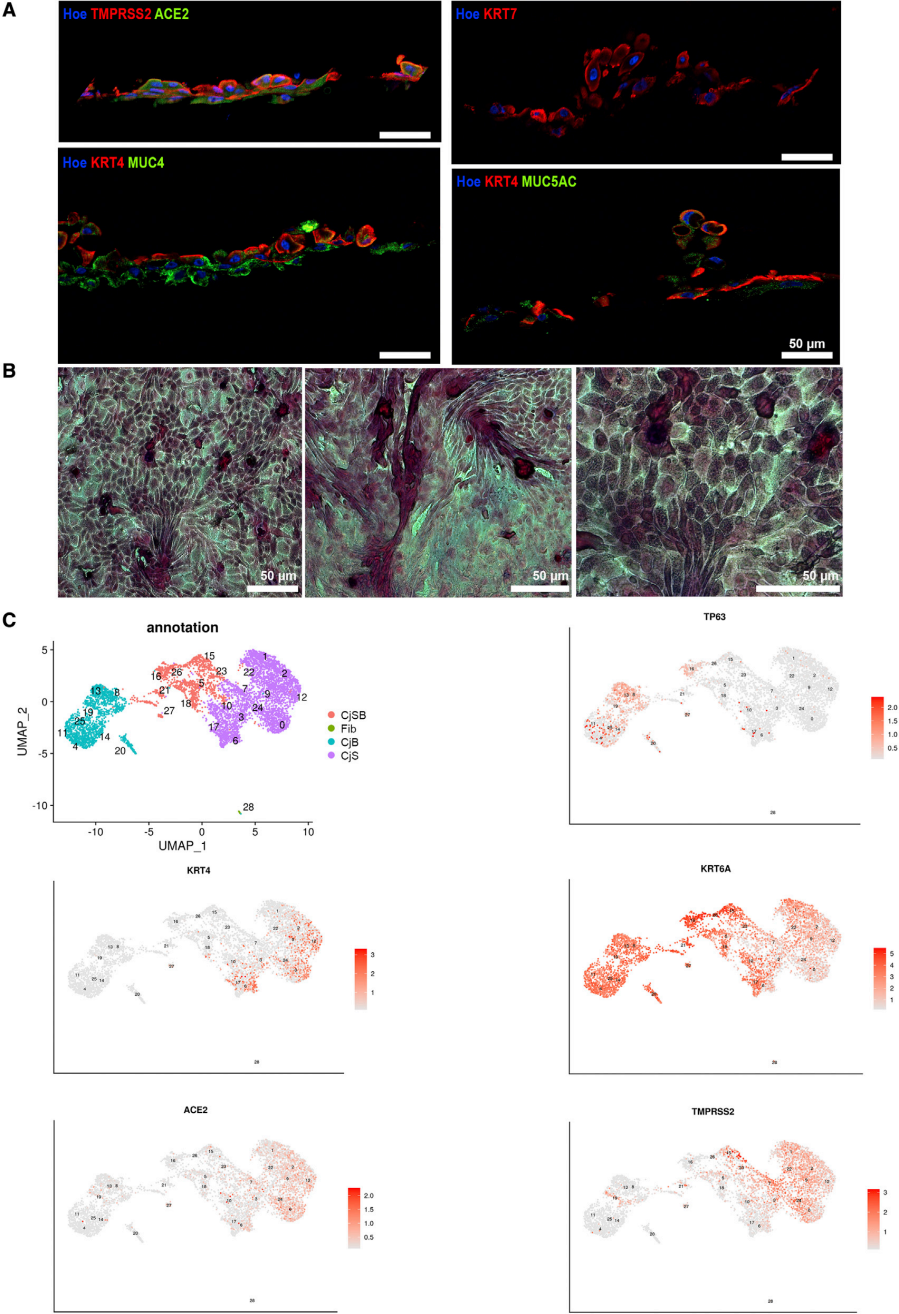
Characterization of ALI conjunctival organotypic culture model at day 30 of differentiation by immunofluorescence and single-cell RNA-seq (A) Immunofluorescence analysis showing co-expression of ACE2 and TMPRSS2 in the superficial layer of the ALI conjunctival organotypic model. KRT7 and KRT4 were predominantly located in the superficial layer, while MUC4 was detected throughout (representative of repeat experiments in three different donor conjunctival ALI cultures). Hoe, Hoescht. Scale bars 50 μm. (B) Presence of neutral mucins (magenta) in whole-mount stains of ALI conjunctival organotypic model (representative of repeat experiments in three different donor conjunctival ALI cultures). Scale bars 50 μm. (C) Uniform manifold approximation and projection (UMAP) visualization of scRNA-seq data from conjunctival ALI cultures (8,202 cells from three different donors) showing the presence of basal (CjB), suprabasal (CjSB), and superficial conjunctival (CjS) epithelium and fibroblasts (Fibs). Expression of key epithelial markers and SARS-CoV-2 entry factors, *ACE2* and *TMPRSS2*, are shown as superimposed single gene-expression plots on the UMAP.

Samples were acquired at days 0, 15, 30, and 50 of differentiation and analyzed by quantitative RT-PCR for expression of several markers (Figure 1B), identified from the single-cell RNA-seq analysis of human ocular surface reported earlier by our group (Collin et al., 2021a). These characterize limbal progenitors and conjunctival basal epithelium (*TP63*), conjunctival epithelium (*KRT13, S100A9*), superficial (*KRT7, MUC4*), and basal-suprabasal conjunctival epithelium (*KRT6A*) (Figure 1C). This analysis demonstrated the persistence of *TP63* throughout the differentiation period and a significant increase in the expression of *KRT13* and *S100A9* from day 0 to 15 (Figure 1C), indicating the onset of differentiation toward conjunctival epithelium. Notably, the expression of *KRT6A* and *KRT7* was increased from day 0 to 15 of differentiation, indicating specification to basal, suprabasal and superficial epithelial conjunctival cells. The expression of superficial conjunctival epithelial markers (*KRT7, MUC4*) was maintained during the differentiation process, while *KRT6A* expression declined, suggesting that the culture conditions were more suited to the development of superficial conjunctival epithelium. The expression of *ACE2* and *TMPRSS2* increased significantly during the first 15 days of differentiation at ALI and was maintained at similar levels to the uncultured conjunctival tissue (Figure 1C). These findings were corroborated by immunofluorescence (IF) analysis, showing co-expression of ACE2 and TMPRSS2, as well as abundant expression of KRT13, and superficial conjunctival epithelium markers KRT4 and KRT7 at both days 15 and 30 of differentiation (Figures 1D and 2A). Importantly, we were able to detect expression of MUC5AC (Figures 1D and 2A) and neutral mucins (Adams and Dilly, 1989) (Figure 2B), suggesting the presence of goblet cells in our ALI conjunctival epithelium organotypic model.

Complementary single-cell RNA-seq analysis of day 30 ALI samples from 3 donors was performed, revealing a predominant superficial conjunctival epithelial cluster (comprising 55.6% of the total cells), alongside basal and suprabasal conjunctival epithelial clusters, comprising 25.9% and 19.1% of the total cells, respectively (Figures 2C; Table S1). Cluster definition was based on the strong expression of *TP63* in the basal conjunctival epithelium and some suprabasal cells (as reported in conjunctiva and respective ALI organotypic cultures) (Lin et al., 2014) and strong *KRT4* expression (Merjava et al., 2011) in the perilimbal superficial conjunctiva (Figure 2C). The suprabasal cluster was defined by expression of *KRT6A* (Figure 2C) and *KRT14* (data not shown), both strongly expressed in the suprabasal conjunctival epithelium but less so in the superficial layers. A much smaller fibroblast cluster was also identified (0.3% of the total cells). Notably, the expression of *ACE2* was highest in the superficial conjunctival epithelium (Figure 2C), while *TMPRSS2* was expressed at a higher level in the superficial but also some basal and suprabasal conjunctival epithelial cells. In total, 26% of cells co-expressed *ACE2* and *TMPRSS2*. To assess the representativeness of the ALI conjunctival model, we correlated expression of the top 2,000 highly variable genes revealing the strongest correlation between the ALI superficial cells *in vitro* and the superficial conjunctival epithelium *in vivo* (Collin et al., 2021b) (correlation coefficient 0.67).

IF analysis at day 75 of differentiation revealed the presence of a multi-layered epithelium, characterized by predominantly apical expression of the superficial conjunctival markers KRT7 and KRT4 and widespread expression of MUC4 (Figure S1A). Single-cell RNA-seq at day 75 of differentiation (Table S1) revealed the presence of similar cell clusters to day 30 as well as superficial epithelial cell expression of *ACE2* and abundant expression of *TMPRSS2* (Figure S1B). By IF analysis, cells co-expressing both ACE2 and TMPRSS2 were found both on the apical and subapical layer of ALI cultures (Figure S1A): those comprised 36% of the total cells analyzed by single-cell RNA-seq.

Transmission electron microscopy (TEM) analysis showed tight junctions between the epithelial cells in addition to apical microvilli. Electron-dense glycocalyx was detected on the surface of microvilli, indicating the formation of a barrier between the cells on the apical surface and the surroundings (Figure S2). Both microvilli and glycocalyx are found on the surface of the conjunctiva and are believed to provide the framework that supports and binds tears, mucus, and immunoglobulins, which have the common function of protecting the eye (Nichols et al., 1983).

Together, these findings demonstrate the establishment of the ALI conjunctival epithelium comprised of basal, suprabasal, and superficial conjunctival cells, which express the typical conjunctival-specific mucins and show the ultrastructural features of normal conjunctival epithelial tissue in humans.

### Conjunctival epithelial ALI organotypic cultures are permissive to SARS-CoV-2 genome expression but are resistant to productive infection

**Figure 3:**
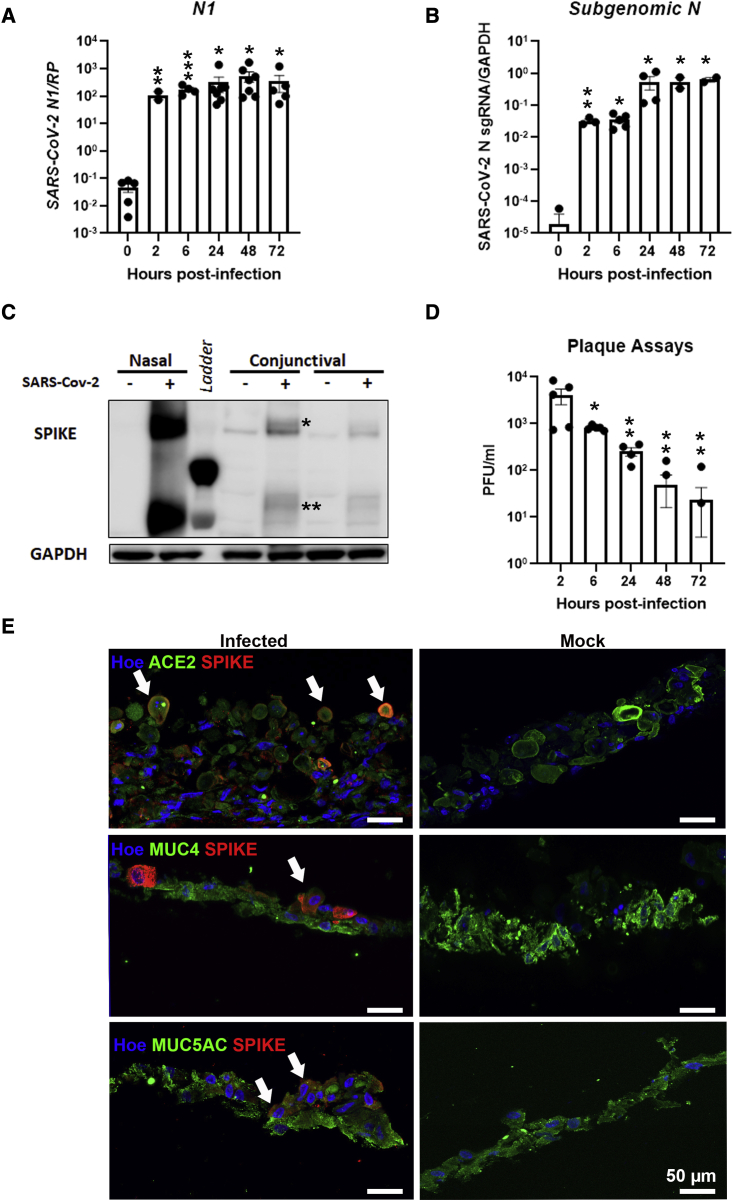
SARS-CoV-2 infection of day 30 human ALI conjunctival organotypic culture (A and B) Quantitative RT-PCR expression of nucleocapsid (*N*) gene (normalized to the housekeeper *RNASEP*) and subgenomic *N* RNA (normalized to *GAPDH*) from 0–72 hpi. Data shown as mean ± SEM, n = 3–7 experimental repeats, 3 different donors, ^∗^p < 0.05, ^∗∗^p < 0.01, ^∗∗∗^p < 0.001, one way ANOVA with Dunnett’s multiple comparisons to 0 hpi. (C) Representative western blot showing the expression of SARS-CoV-2 spike (S; shown by asterisk) and cleaved S2 protein expression (shown by two asterisks) in the nasal and conjunctival ALI organotypic culture models. GAPDH was used as loading control (representative of repeat experiment in 3 donors). (D) Release of infectious viral particles was determined by plaque assay using apical washings from 2–72 hpi. Data shown as mean ± SEM, n = 2–5, 3 different donors, ^∗^p < 0.05, ^∗∗^p < 0.01, one way ANOVA with Dunnett’s multiple comparisons to 2 hpi. (E) Immunofluorescence analysis showing the presence of infected cells marked by ACE2 and S co-expression. A few mucin-secreting cells are also infected by SARS-CoV-2, as shown by co-expression of MUC4 and MUC5AC with S (white arrows). A panel of mock-infected cells is shown on the right-hand side panel, and white arrows indicate MUC5AC-secreting cells (representative of repeat experiment in 3 donors). Hoe, Hoescht. Scale bars 50 μm.

To assess the permissiveness of conjunctival epithelial cells to infection, conjunctival ALI organotypic cultures were inoculated with a clinical isolate of SARS-CoV-2 (BetaCoV/England/2/2020, multiplicity of infection [MOI] = 0.5) at the apical surface for 2 h, the inoculum was removed, and the infection was assessed regularly for 72 h post-infection (hpi). This MOI was selected as it is consistent with previous studies of SARS-CoV-2 infectivity in ocular cultures (Miner et al., 2020; Eriksen et al., 2021). Expression of SARS-CoV-2 nucleocapsid (*N*) genomic and subgenomic *N* RNA was detected in cell lysates from 2–72 hpi, suggesting permissiveness to SARS-CoV-2 entry and genome replication. However, there was no significant increase in viral RNA expression over time (Figures 3A and 3B), in contrast to nasal epithelial ALI cultures (Hatton et al., 2021), suggesting a relative resistance to productive replication. Consistent with this, SARS-CoV-2 subgenomic *N* RNA abundance at 72 hpi was at least two orders of magnitude lower than in nasal epithelium ALI cultures infected at a similar MOI (0.1) (Figure S3B). Consequently, although SARS-CoV-2 spike (S) protein was detected by western blot at 72 hpi, the expression was substantially lower than infected nasal epithelium ALI cultures (Figure 3C). IF analyses at 48 hpi corroborated expression of S protein in conjunctival epithelial cell types, including the mucin secreting cells (Figure 3E). To address the permissiveness of conjunctival cells to productive infection, we measured the release of infectious particles by plaque assays on superficial washes over time. This analysis showed a continuous decline in infectious particle detection from 2 to 72 hpi, indicating that the conjunctival epithelium ALI cultures did not support productive infection (Figure 3D). Consistent with these findings, we were unable to identify virion-like structures by TEM analysis at 48 hpi. Together, these data demonstrate that while conjunctival epithelial cells are permissive to SARS-CoV-2 entry and genome replication, they are unable to support productive infection, extending recent findings in an alternative conjunctival model (Singh et al., 2021).

### Transcriptional response of conjunctival epithelial cells to SARS-CoV-2 infection

**Figure 4:**
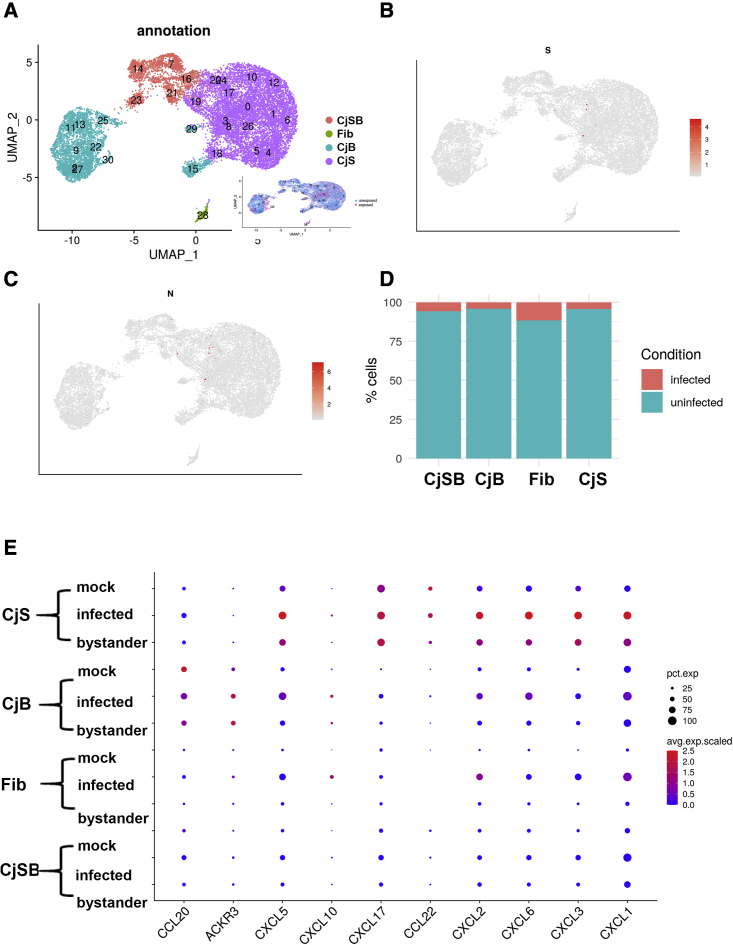
Single-cell RNA-seq analyses at 24 hpi reveal broad but low tropism of SARS-CoV-2 in the ALI conjunctival organotypic culture model (A) UMAP visualization of scRNA-seq data from mock and infected conjunctival ALI cultures (15,821 cells from three different donors, mock and SARS-CoV-2 infected) showing the presence of CjB, CjSB, and CjS epithelium and Fibs. A smaller UMAP on the right bottom corner shows the overlap between SARS-CoV-2-exposed and unexposed cultures. (B and C) Expression of S and N transcripts shown as superimposed single gene-expression plots on the UMAP. (D) Relative proportion of infected cell types (CjB, CjSB, CjS, Fibs) based on expression of any viral transcript. (E) Dot plot demonstrating expression of key chemokine marker upregulated in response to SARS-CoV-2 infection in all cell types, with intensity demonstrated by color and size of the dot representing the proportion of cells expressing the marker.

To further assess SARS-CoV-2 cell-type-specific tropism, we performed single-cell RNA-seq (scRNA-seq) at 24 hpi, which represents an early stage in the infection process and a peak of viral gene expression. 15,821 cell transcriptomes from three infected (MOI = 0.5) and three uninfected ALI organotypic cultures were integrated and analyzed. Cellbender (v.0.2) (Fleming et al., 2019) was used to remove potentially misleading background from ambient RNA. This analysis defined four cell clusters corresponding to superficial conjunctival epithelium (59.1%), basal conjunctival epithelium (26.3%), suprabasal conjunctival epithelium (13.5%), and fibroblasts (1%) (Figure 4A; Table S2). Viral transcripts were identified in all of these cell types, albeit at a low percentage (4.1% of basal, 5.7% of suprabasal, 4.4% of superficial conjunctival epithelium, and 11.6% of fibroblasts) (Figures 4B and 4C). This was substantially lower than nasal epithelial cells, where viral transcripts were detected in 25%–80% of individual cell types using a comparable scRNA-seq methodology (Hatton et al., 2021). Together, these data suggest that SARS-CoV-2 is capable of infecting all cell types of the conjunctival ALI model, but at low efficiency, consistent with our previous findings.

**Figure 5:**
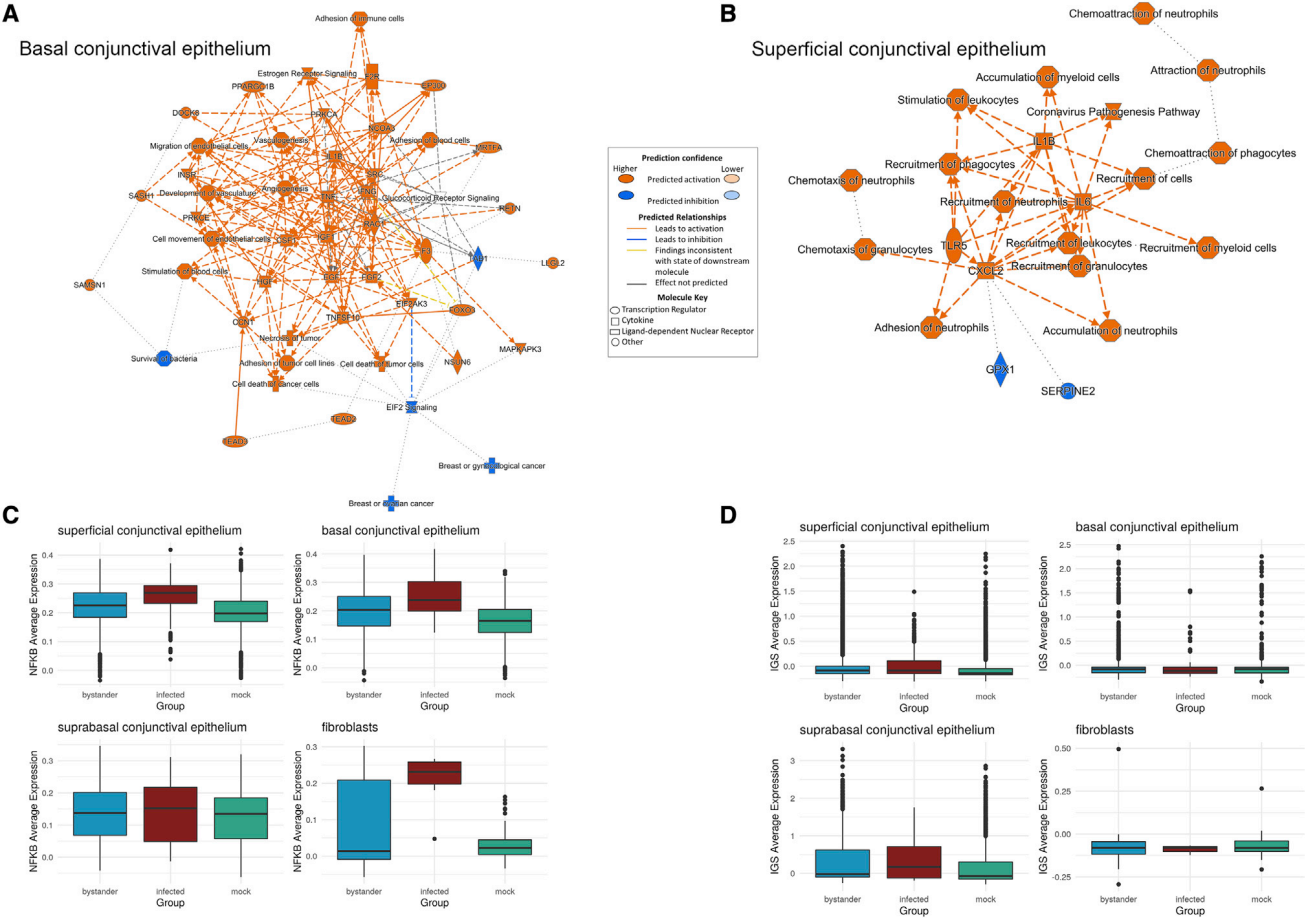
SARS-CoV-2 infection in conjunctival cells is characterized by robust autocrine and paracrine NF-κB activity (A and B) Representative network analysis of predicted regulators in the SARS-CoV-2-infected cells in the CjB (A) and CjS (B) epithelial cells. Differentially expressed genes between infected and mock cells within the CjB and CjS epithelium cluster were generated using the Seurat *FindMarkers* function. IPA upstream regulator analysis was used to predict upstream transcriptional regulators from this gene list, using the Ingenuity Knowledge Base to create mechanistic networks. (C and D) NF-κB target (C) and IFN-stimulated gene (ISG) expression (D) in infected, bystander, and mock in the CjB, CjSB, and CjS epithelium and fibroblasts. Gene set scores greater than zero suggest expression levels higher than background gene expression. The bottom and the top of the boxes correspond to the 25th (Q1) and 75th (Q3) percentiles, and the internal band is the 50th percentile (median). The plot whisker minimum is calculated as Q1 -1.5 × interquartile range (IQR) and the maximum as Q3 +1.5 × IQR. IQR, interquartile range. Outside points correspond to potential outliers.

Given these findings, we next sought to assess the cell-type-specific host cell response to SARS-CoV-2 infection, hypothesizing that a more robust innate immune response might account for the reduced permissiveness of conjunctival epithelial cells to SARS-CoV-2. We performed differential gene expression (DE) analysis for each cell type (adjusted p < 0.05), defining three experimental conditions: SARS-CoV-2 infected (as defined by detectable expression of at least one viral gene), SARS-CoV-2 exposed but uninfected (bystander cells), and unexposed (mock-infected cells). DE analysis of SARS-CoV-2-infected to mock cells (Table S3) revealed the significant upregulation of several chemokines (*CXCL1*, *CXCL6*, *CXCL3*, *CXCL5*, *CXCL8*, *CXCL2*, *CXCL17*) in the infected and bystander superficial conjunctival epithelium (Figure 4E) and, to a lesser extent, in the basal conjunctival epithelium (*CXCL5*, *CXCL17*, *CXCL10*, *CXCL3*, *CXCL6*, *CXCL2*, *CXCL1*), corroborating recent findings reported by Eriksen et al. in SARS-CoV-2-infected scleral cells (Eriksen et al., 2021). Importantly, several tumor necrosis factor (TNF)- and interleukin-1 (IL-1)-regulated genes (*C3*, *CD55*, *CD47*) were upregulated in infected basal conjunctival epithelial cells (Table S3), consistent with the predicted activation of various pattern recognition and NF-κB-dependent signaling pathways by ingenuity pathway analysis (IPA) (Table S4; Figure 5A). Similarly, TNF- and IL-1-regulated genes were observed in SARS-CoV-2-infected superficial conjunctival epithelial cells (Table S4; Figure 5B) alongside the upregulation of *IL-6*, indicative of a robust NF-κB response. To confirm the NF-κB response, ALI cultures were pre-incubated with the IKKβ inhibitor, BI605906 (10 μM), which blocks NF-kB activation, prior to SARS-CoV-2 infection. This significantly reduced the expression of *CXCL8* and *TNF* (Figure S3D), confirming their NF-κB dependence, yet had no impact on expression of SARS-CoV-2 *N* gene at 24 hpi (Figure S3C), suggesting that NF-κB did not substantially impact viral genome expression under these conditions.

Given the prominence of NF-κB-driven transcription in infected cells, we undertook gene set enrichment analysis of NF-κB target genes, finding that expression was upregulated in infected cells compared with mock or bystander cells (Figure 5C). Notably, there was no widespread induction of antiviral IFN signaling, identified by expression of IFN-stimulated genes (ISGs) (Figure 5D). Consistent with this, analysis of the bulk transcriptional response by RT-PCR identified no significant increase in antiviral ISGs such as *IFITM1-3* up to 72 hpi (Figure S3E). Indeed, there was evidence of downregulation of certain ISGs, including *IFI6* (also known as IFI-6-16) in superficial conjunctival epithelial cells (Tables S3), indicating evasion of an IFN response in infected cells by SARS-CoV-2. Significantly enriched signaling pathways and biological processes in conjunctival superficial and basal epithelial cells included EIF2 stress, glucocorticoid receptor signaling, the coronavirus pathogenesis pathway, complement system, mitochondrial dysfunction, and oxidative phosphorylation (Table S5), corroborating recent findings reported by the comprehensive human-SARS-CoV-2 interactome (Kumar et al., 2020).

A more muted transcriptional response to SARS-CoV-2 infection was observed in suprabasal conjunctival cells and fibroblasts, with only 6 and 4 differentially expressed genes being identified, respectively, apart from the viral transcripts (Table S3).

### Robust paracrine signaling in response to SARS-CoV-2 infection

**Figure 6:**
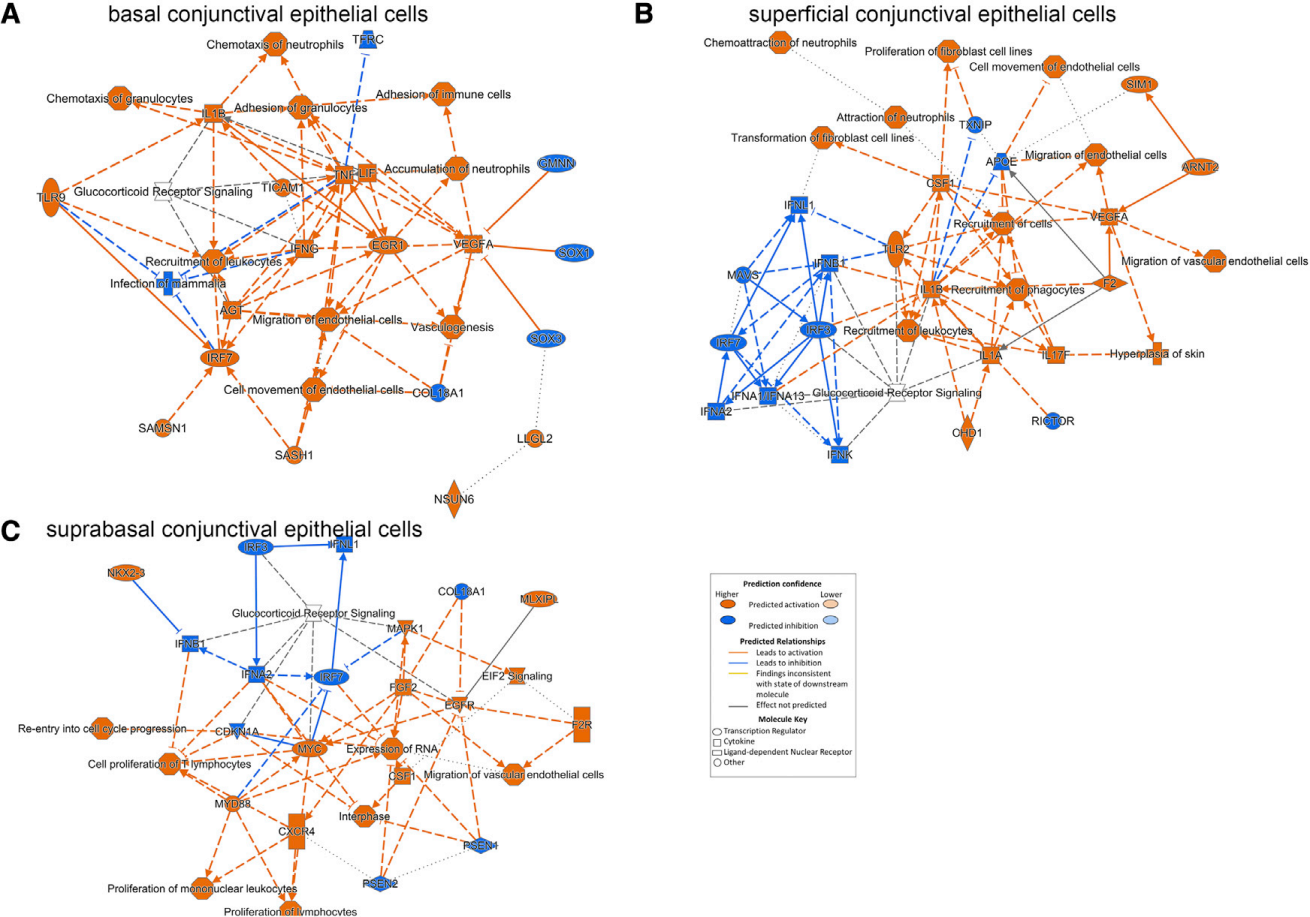
Evasion of IFN response in infected conjunctival epithelial cells by SARS-CoV-2 (A–C) Representative network analysis of predicted regulators in the bystander cells in the CjB (A), superficial (B), and CjSB (C) epithelial cells. Differentially expressed genes between bystander and mock-infected cells within the CjB, CjSB, and CjS epithelial cells were generated using the Seurat *FindMarkers* function. IPA upstream regulator analysis was used to predict upstream transcriptional regulators from this gene list, using the Ingenuity Knowledge Base to create mechanistic networks.

We next asked whether there was evidence of a paracrine immune signaling response to SARS-CoV-2 in bystander cells, which are exposed to factors produced by SARS-CoV-2-infected cells but are not themselves infected. The analyses of bystander versus mock-infected cells revealed several DE genes in all four cell types (Table S6), suggestive of a robust paracrine response. In general, this response mirrored that of infected cells in that it was dominated by NF-κB signaling without evidence of an antiviral IFN response. Several chemokines were upregulated in bystander conjunctival superficial and basal epithelial cells (Figure 4E), consistent with predicted activation of upstream regulators including IL-1 and TNF (Figures 6A and 6B; Table S7). These data indicate that SARS-CoV-2 infection triggers proinflammatory NF-κB signaling in bystander conjunctival superficial and basal epithelial cells, corroborated by activation of NF-κB target genes in bystander versus mock-infected cells (Figures 6A and 6B). Assessment of context-specific ISG expression identified no evidence of induction of an IFN response in either infected or bystander superficial and basal conjunctival epithelial cells (Figure 5D).

Increased expression of genes involved in keratinisation (*SPRR3* in basal) and various keratins (*KRT17*, *KRT5*, *KRT14*, *KRT6A*, and *KRT6B* in the superficial conjunctival epithelium; Table S6) was observed in bystander cells, indicative of a wider keratinization occurring in the conjunctival epithelium upon SARS-CoV-2 infection. An increase in expression of genes involved in keratinization has been reported in the tears collected from patients with COVID-19 (Mastropasqua et al., 2021). These findings are interesting and might suggest a potential molecular pathomechanism underlying the keratoconjunctivitis reported in some of the patients with COVID-19 (Loffredo et al., 2021; Al-Namaeh, 2021; Ozturker, 2021).

Notably, in both superficial and suprabasal conjunctival epithelial cells, there was some evidence of suppression of paracrine type I and III IFN signaling (Figures 6B and 6C), extending recently published findings (Xia et al., 2020; Park and Iwasaki, 2020; Fung et al., 2021; Decker, 2021), suggestive of viral evasion of IFN signaling in bystander cells. It is not clear whether this is due to a paracrine effect of SARS-CoV-2 itself or an indirect consequence of other host signals, such as TNF, which is recognized to suppress IFN responses in certain contexts (Banchereau et al., 2004).

### Transcriptional differences between the nasal and conjunctival epithelial response to SARS-CoV-2

**Figure 7:**
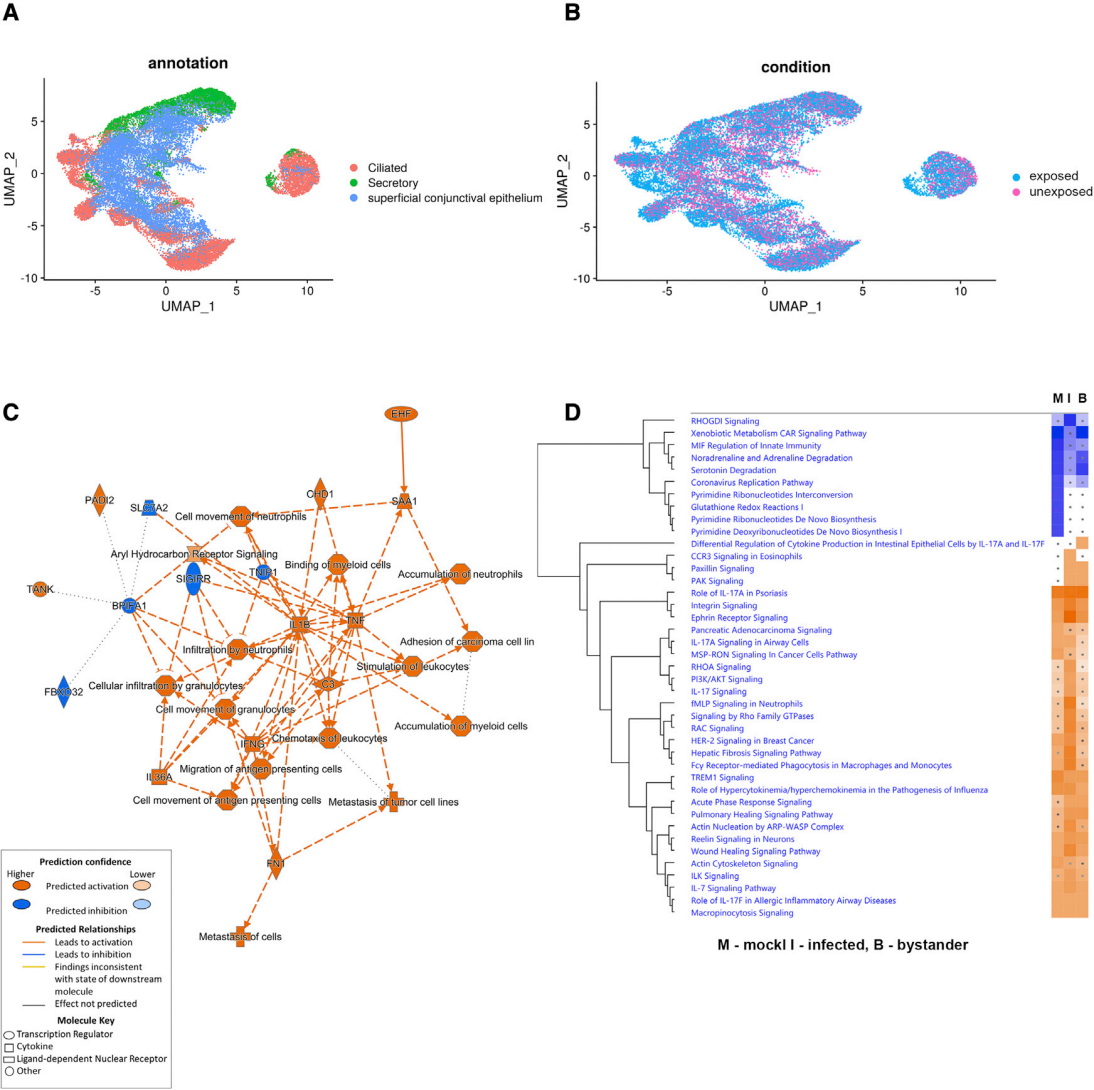
Transcriptomic comparison of conjunctival and nasal epithelium response to SARS-CoV-2 infection (A and B) UMAPs showing the integration of single-cell RNA-seq data of CjS and nasal secretory and ciliated epithelial cells exposed and unexposed to SARS-CoV-2. (C) Differentially expressed genes between unexposed (mock) CjS and nasal secretory and ciliated epithelial cells were generated using the Seurat *FindMarkers* function. IPA upstream regulator analysis was used to predict upstream transcriptional regulators from this gene list, using the Ingenuity Knowledge Base to create mechanistic networks. (D) An IPA comparison analysis of canonical pathways identified by a comparison between CjS and secretory and ciliated nasal epithelial cells within mock (M), bystander (B), and infected (I) groups. The results are filtered to show any significant pathways with an absolute *Z* score of 2 or more. Orange represents pathways with a positive *Z* score that are activated, and blue indicates negative *Z* scores that are deactivated in CjS compared with secretory and ciliated nasal epithelial cells. A dot indicates pathways with an absolute *Z* score of <2.

We previously reported that nasal epithelial cells were permissive to productive SARS-CoV-2 infection (Hatton et al., 2021), in contrast to conjunctival epithelial cells. To explore cell-type-specific transcriptional signatures that might underlie this differential viral susceptibility, scRNA-seq data generated from superficial conjunctival epithelial cells were compared with published secretory and ciliated nasal epithelial cell datasets (all at 24 hpi; Hatton et al., 2021). Following data integration (Figures 7A and 7B), infected, bystander, and mock cells were identified and subjected to DE gene analysis (Table S8) followed by pathway enrichment and identification of upstream regulators (Tables S9 and S10). We first examined mock-infected cells to identify differences in the resting state. Several TNF- and IL-1B-stimulated genes such as chemokines (*CXCL20*, *CXCL3*, *CXCL5*, *CXCL6*, *CXCL8*) and S100 calcium-binding proteins (*S100A7*, *S100a8*, *S100A9*) were significantly upregulated in uninfected conjunctival superficial epithelial cells (Table S8), implying a more efficient basal engagement of the inflammatory response in conjunctival cells (Figure 7C). Although there was no significant difference in expression of viral entry receptors *ACE2* or *TMPRSS2*, *TMPRSS4*, a protease reported to mediate SARS-CoV-2 entry in enterocytes alongside TMPRSS2 (Zang et al., 2020), was expressed at a higher level in nasal secretory and ciliated epithelial cells, which might be relevant to their greater permissiveness. In analysis of both infected and bystander conjunctival epithelial cells compared with nasal cells, p21-activated kinase (PAK) signaling was significantly upregulated (Figure 7D). PAK1 has been shown to promote NF-κB responses, and this seems to align with prior observations of autocrine and paracrine NF-κB activation in SARS-CoV-2-exposed conjunctival cells.

## Discussion

The human ocular epithelium is continuously exposed to infectious droplets and contaminated fomites. Of the three epithelial segments that comprise the ocular surface epithelium, corneal epithelium has been shown to be resistant to SARS-CoV-2 infection (Miner et al., 2020), while the adjacent limbal epithelium, which harbors the corneal epithelial stem cells, expresses ACE2 and TMPRSS2 at high levels and appears permissive to viral infection (Eriksen et al., 2021). Yet, few studies have addressed the permissiveness of the conjunctival epithelium, the largest exposed component of the human ocular surface. A recent study showed that conjunctival epithelium could be infected with SARS-CoV-2; however, the ability of this tissue to sustain productive replication, which is highly relevant to its place as a potential entry route for the virus, and the response of individual conjunctival epithelial cell types to infection remain unresolved (Singh et al., 2021).

Conjunctival and corneal organotypic cultures and cultured epithelial monolayers have traditionally been used to examine the capacity of virus infection and replication in the human ocular surface. The *ex vivo* organotypic cultures are limited in numbers, and the monolayer cultures do not capture the involvement of ocular surface mucins or the various cell-type interactions, which are necessary for understanding the innate immune response in viral infection dynamics. The ALI organotypic conjunctival model reported in this study overcomes these limitations, as it can be generated in large numbers and comprises all the key conjunctival cell types, including mucin-secreting cells, which play an important role in the ocular surface defense against viruses (Mantelli and Argüeso, 2008). Importantly, glycocalyx, a layer of glycolipids and glycoproteins, forming a barrier between the apical surface of the ALI model and the surrounding area, was observed, mimicking the native barrier of conjunctival epithelium. Notably, this model lacks the rapid turnover of secreted mucins due to lack of tears and reflex blinking that, together with the mucins, help with clearance of allergens and pathogens (Popov, 2020).

Consistent with our previous single-cell studies showing expression of relevant entry receptors *ACE2* and *TMPRSS2,* in approximately 6.6% of conjunctival epithelial cells *ex vivo* (Collin et al., 2021b), SARS-CoV-2 infection of this ALI conjunctival epithelial model indicated broad but relatively inefficient infection of the various cell types by SARS-COV-2. Importantly, our data showed no evidence of productive replication in the conjunctival epithelium, consistent with recent findings in the corneal epithelium (Miner et al., 2020). Reasons for this apparent post-entry restriction to replication in different ocular surface cell types remain to be defined and did not appear to relate to the preferential engagement of an antiviral IFN response but do contrast with permissiveness of the conjunctival epithelium to other respiratory viruses (Belser et al., 2012). These data are consistent with the apparently paradoxical detection of SARS-CoV-2 nucleic acid or protein in postmortem tissue samples but the relatively infrequent detection of viral RNA in tears or conjunctival swabs of patients with COVID-19. These data are also consistent with the low incidence of clinical conjunctivitis in patients with SARS-CoV-2 infection and indicate a relatively low risk of SARS-CoV-2 transmission from conjunctival transplantation.

Using scRNA-seq analysis, we observed an increase in proinflammatory cytokine expression in SARS-CoV-2-infected superficial and basal conjunctival cells and, to a lesser extent, in basal epithelial cells. Proinflammatory cytokine expression is driven by the NF-κB, a family of transcription factors, consisting of RelA, RelB, NF-κB1, NF-κB2, and c-Rel homo/heterodimers with RelA or RelB. A very recent study has shown that ORF3a, ORF7a, and N proteins of SARS-CoV-2 act as NF-κB activators, with ORF7a being the most potent NF-κB inducer and proinflammatory cytokine producer (Su et al., 2021). Multiple pieces of evidence point to activation of NF-κB in ocular surface cells upon infection with adenovirus (Rajaiya et al., 2009), influenza A viruses (Belser et al., 2011), and respiratory syncytial virus (RSV) (Bitko et al., 2004). Together, these data indicate that the NF-κB activation we and others (Eriksen et al., 2021) have observed is not specific to SARS-CoV-2 but is a general response to viral infection at the ocular surface (Lan et al., 2012). Interestingly, there was also evidence of activation of NF-κB signaling in resting conjunctival epithelial cell types compared with more permissive nasal secretory or ciliated epithelial cells. However, we found no evidence that this response was an important determinant of viral permissiveness since there was no enhancement of conjunctival cell infection in the context of NF-κB blockade. Further work is needed to determine the mechanism(s) underlying the apparent post-entry restriction to productive SARS-CoV-2 infection in conjunctival epithelial cells.

In conclusion, the data presented herein show that conjunctival epithelium is permissive to SARS-CoV-2 infection but is without evidence of productive viral replication. This study was performed in organotypic models derived from three different patients, with scRNA-seq data obtained from the peak infection interval. Future work should assess changes in transcriptome of each conjunctival cell type at frequent intervals after infection and in a larger number of donors to get deeper insights into the refractory nature of these cell to viral propagation.

## Experimental procedures

### Human tissue donation

Adult human eyes from three female donors of 52, 78, and 80 years old were donated for research following informed consent. All tissue was provided by NHS Blood and Transplant Tissue and Eye Services or the Newcastle NHS Trust following ethical approval (18/YH/04/20).

### *Ex vivo* expansion of conjunctival epithelial cells

Human conjunctival epithelial cell expansion on mitotically inactivated 3T3 feeder cells was performed using methods described previously for limbal epithelial cell expansion (Collin et al., 2021a). Cell colonies with typical epithelial morphology started to appear after 4–7 days and were cultured until they became subconfluent. Following this, 3T3 feeder cells were detached and removed using 0.02% EDTA (Lonza, Switzerland), and subconfluent primary cultures were dissociated with 0.5% trypsin-EDTA (Santa Cruz, CA, USA) to single-cell suspension and passaged at a density of 6 × 10^3^ cells/cm^2^.

### Generation of ALI conjunctival and nasal organotypic culture model

ALI differentiation of conjunctival epithelial cells was performed using a method developed for differentiation of lung epithelial basal cells described by Djidrovski et al. (2021). 250,000 epithelial cells were detached from feeders as described above and seeded onto Matrigel-coated 24-well inserts (ThinCerts, Greiner Bio-One) and fed apically and basally with BEGM Bronchial Epithelial Cell Growth Medium Bullet Kit (Lonza) supplemented with 10 μM Y26732 (Sigma Aldrich) and incubated for 48–72 h until confluent. Once confluent, the apical medium was removed, and the cells were basally fed with PneumaCult media (StemCell Technologies) for up to 75 days. The nasal organotypic culture model was performed as described in Hatton et al. (2021).

### Infection of conjunctival ALI cultures with SARS-CoV-2

A clinical isolate from Public Health England of SARS-CoV-2 (BetaCoV/England/2/2020) virus was propagated once in Vero E6 cells. The same viral stock was used for all experiments. As SARS-CoV-2 is a hazard group 3 pathogen (Advisory Committee on Dangerous Pathogens, UK), all infection experiments were performed in a dedicated containment level 3 (CL3) facility by trained personnel. Infections of conjunctival ALI cultures were performed at day 30 of differentiation as previously described (Hatton et al., 2021). The apical washes were collected in 1x phosphate-buffer solution (1xPBS) at 2, 6, 24, 48, and 72 hpi for plaque assays, which were performed as described in Hatton et al. (2021).

## Author contributions

R.M.J., C.F.H., J.S.S., and M.G.: experimental design and performance, data acquisition and analysis, and contributed to manuscript writing and figure preparation; J.C.: scRNA-seq data acquisition and fund raising; E.S., R.H., J.M.C., and M.H.: scRNA-seq acquisition and deposition; B.V. and I.J.H.: experimental design and performance and data acquisition and analysis; T.D., H.-S.M., and B.W.: TEM data acquisition and analysis; P.R., M.H., S.H., C.M.A.K., C.W., and M.B.: provided tissue, reagents, expertise, facilities, and funding and supervised research; F.F. and L.A.: study design and fund raising; R.Q., C.J.A.D., and M.L.: study design, data analysis, manuscript writing, fund raising, and supervised research.

## Conflicts of interest

The authors state no conflicts of interest.

## Data Availability

The scRNA-seq data datasets produced in this study are deposited in the Gene Expression Omnibus. The accession number is GEO: GSE191232. Analysis scripts and codes are available at https://github.com/RachelQueen1/conjunctival_covid.

## Supplemental information

Document S1. Supplemental experimental procedure, Figures S1–S3, and Table S11

Table S1. A full list of highly and differentially expressed genes between the clusters identified in the ALI conjunctival organotypic culture at days 30 and 75 of differentiation

Table S2. A full list of highly and differentially expressed genes between the clusters identified in the SARS-CoV-2-infected and mock ALI conjunctival organotypic culture at day 30 of differentiation

Table S3. A list of differentially expressed genes (adjusted p < 0.05) between infected and mock condition in all cell types

Table S4. List of significant regulators of gene expression in the infected cells (versus mock) in all conjunctival epithelial cell types

Table S5. List of IPA enriched pathways in infected cells (versus mock) in all cell types

Table S6. A list of differentially expressed genes (adjusted p < 0.05) between bystander and mock condition in all cell types

Table S7. List of significant regulators of gene expression in the bystander cells (versus mock) in all cell types

Table S8. A list of differentially expressed genes (adjusted p < 0.05) between superficial conjunctival and secretory and ciliated nasal epithelial cells in the infected, bystander, and mock groups

Table S9. List of IPA enriched pathways in superficial conjunctival (versus secretory and ciliated nasal epithelial) cells in the infected, bystander, and mock groups

Table S10. List of significant regulators of gene expression in the superficial conjunctival (versus secretory and ciliated nasal epithelial) cells in the infected, bystander, and mock groups

Document S2. Article plus supplemental information
