# Supplementary material for: Conjunctival epithelial cells resist productive SARS-CoV-2 infection
Source: Stem Cell Reports. 2022 Jun 23;17(7):1699–713. doi: 10.1016/j.stemcr.2022.05.017 (PMC9222349; doi:10.1016/j.stemcr.2022.05.017)
Supplement: Document S1. Supplemental experimental procedure, Figures S1–S3, and Table S11 [file mmc1.pdf]

Stem Cell Reports, Volume *17*

Supplemental Information

Conjunctival epithelial cells resist productive SARS-CoV-2 infection

Robert M. Jackson, Catherine F. Hatton, Jarmila Stremenova Spegarova, Maria Georgiou, Joseph Collin, Emily Stephenson, Bernard Verdon, Iram J. Haq, Rafiqul Hussain, Jonathan M. Coxhead, Hardeep-Singh Mudhar, Bart Wagner, Megan Hasoon, Tracey Davey, Paul Rooney, C.M. Anjam Khan, Chris Ward, Malcolm Brodlie, Muzlifah Haniffa, Sophie Hambleton, Lyle Armstrong, Francisco Figueiredo, Rachel Queen, Christopher J.A. Duncan, and Majlinda Lako

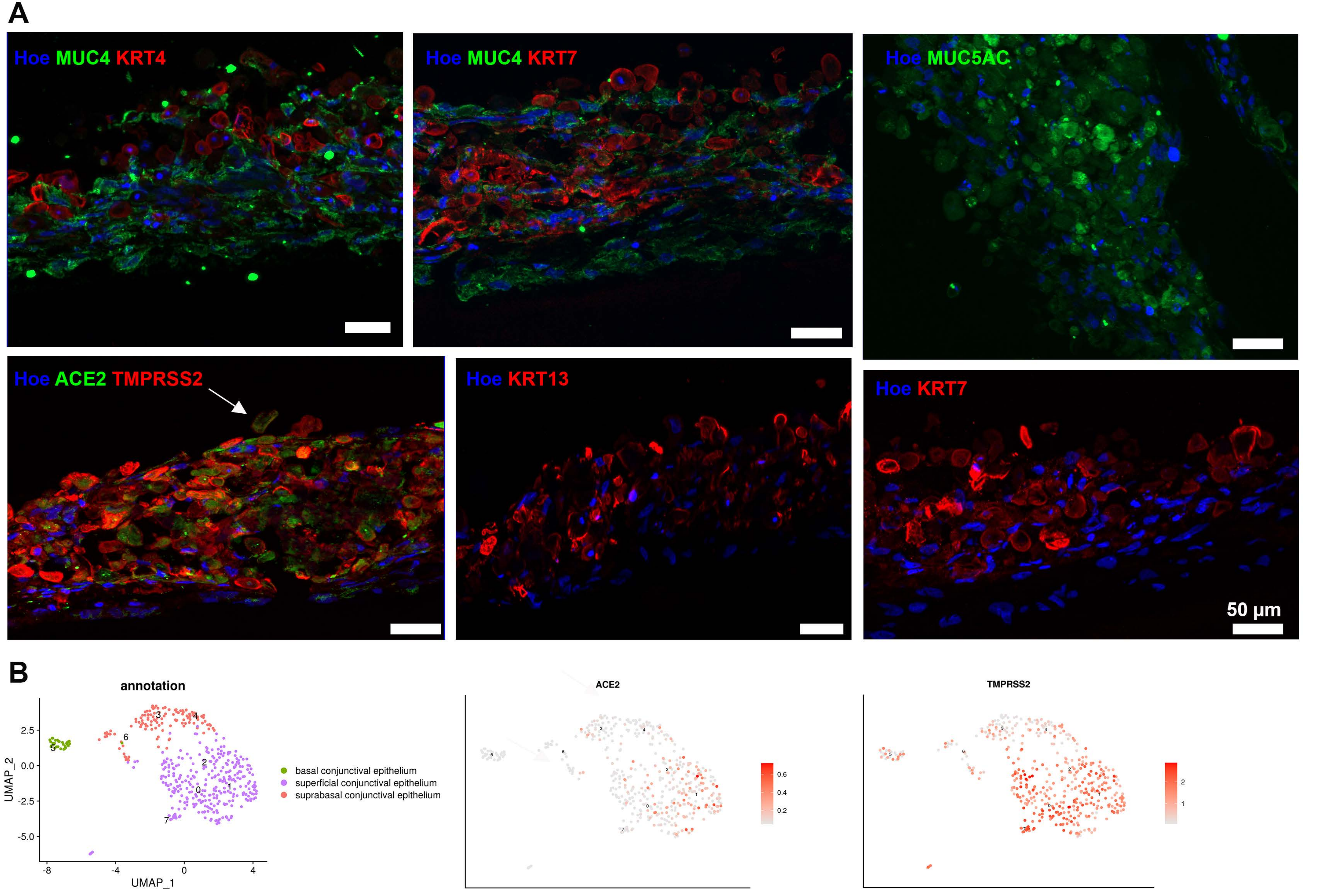

A
Hoe MUC4 KRT4
Hoe MUC4 KRT7
Hoe MUC5AC
Hoe ACE2 TMPRSS2
Hoe KRT13
Hoe KRT7
50 µm
B
annotation
basal conjunctival epithelium
superficial conjunctival epithelium
suprabasal conjunctival epithelium
UMAP_1
UMAP_2
ACE2
TMPRSS2

Figure S1

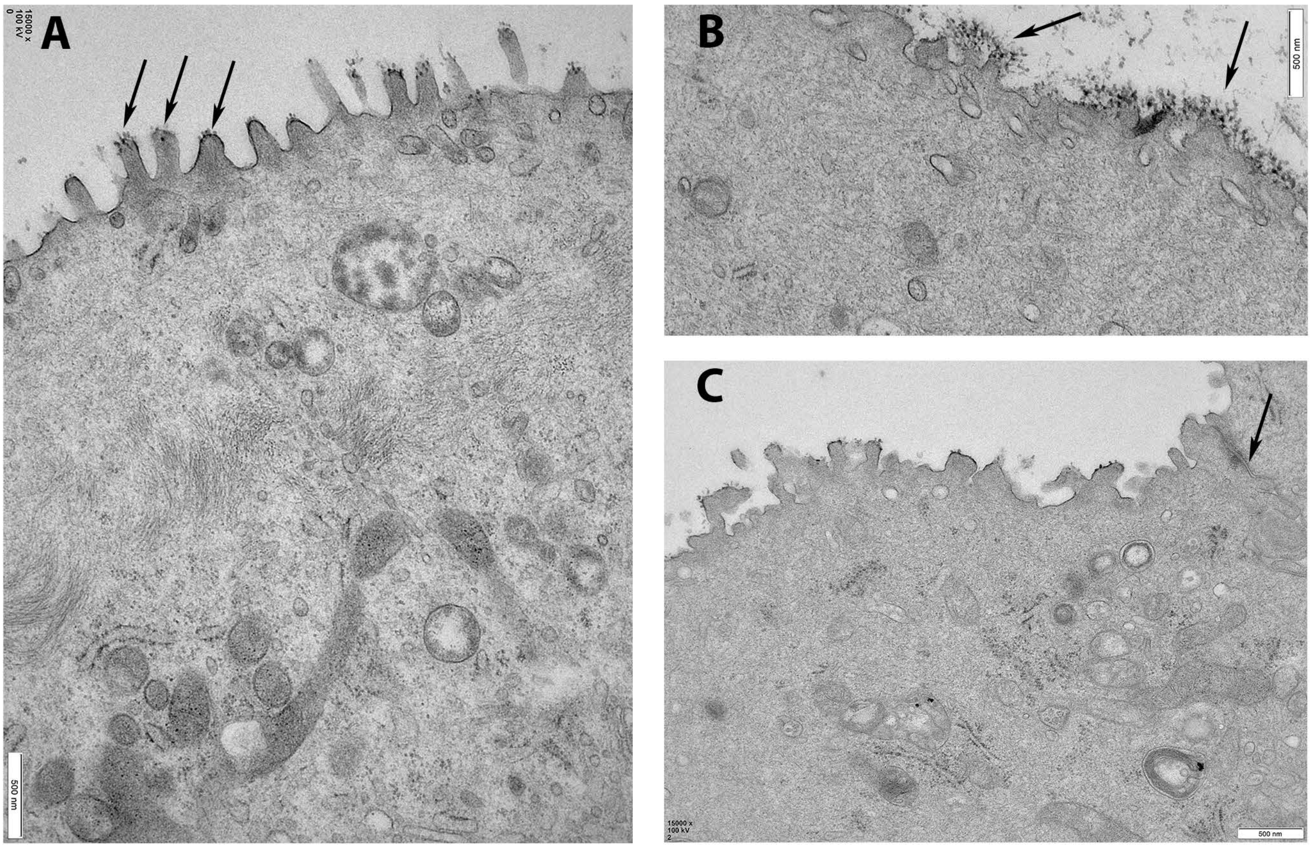

Figure S2

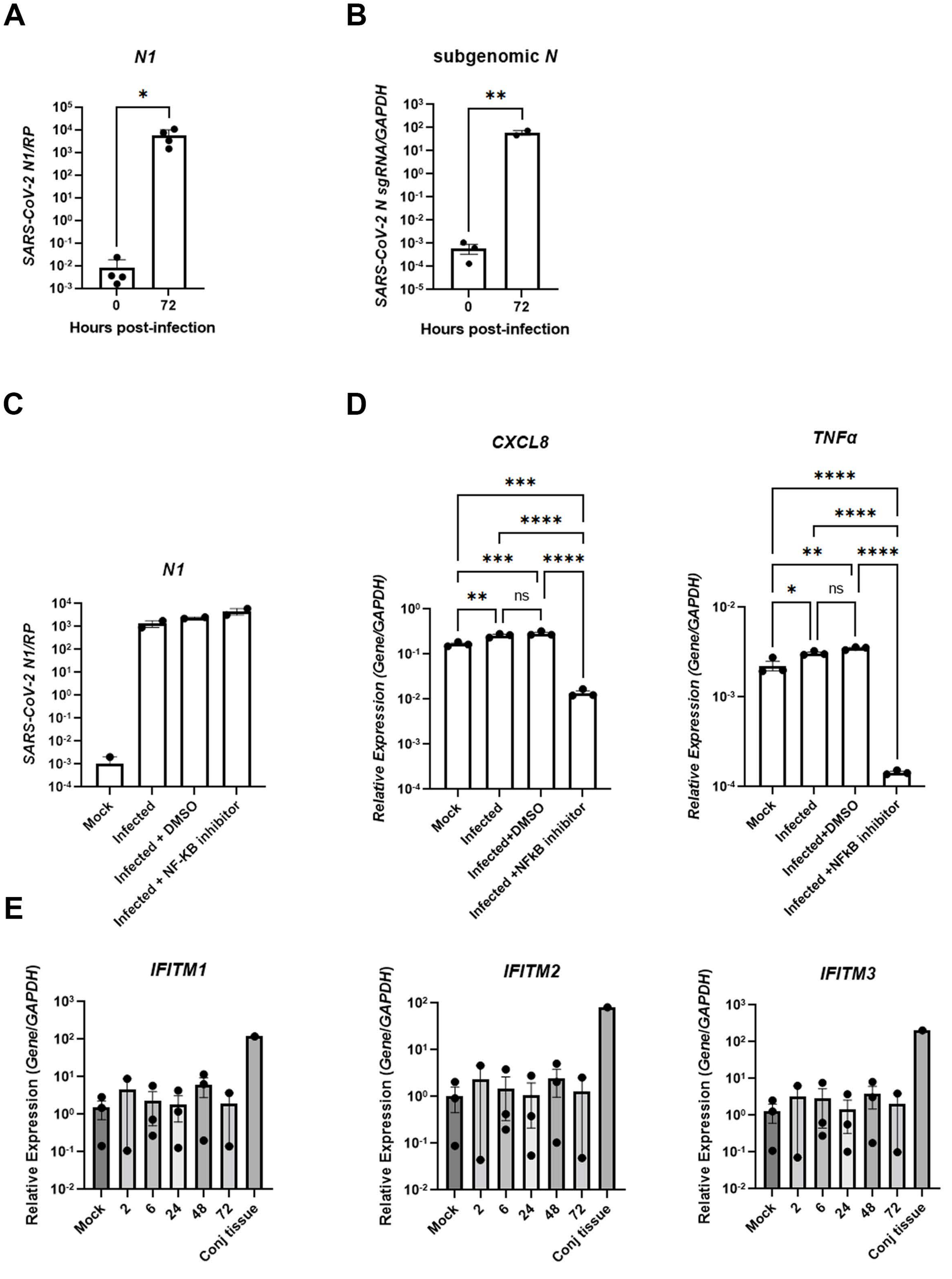
A
N1
SARS-CoV-2 N1/RP
Hours post-infection
B
subgenomic N
SARS-CoV-2 N sgRNA/GAPDH
Hours post-infection
C
N1
SARS-CoV-2 N1/RP
Mock
Infected
Infected + DMSO
Infected + NF-KB inhibitor
D
CXCL8
TNFα
Relative Expression (Gene/GAPDH)
Mock
Infected
Infected+DMSO
Infected +NFkB inhibitor
E
IFITM1
IFITM2
IFITM3
Relative Expression (Gene/GAPDH)
Mock
Conj tissue

Figure S3

Supplementary Information

Figure S1. Characterisation of ALI conjunctival organotypic culture model at day 75 of differentiation by immunofluorescence and single cell RNA-Seq. A) Immunofluorescence analysis showing co-expression of ACE2 and TMPRSS2 (white arrow) in the superficial layer of the ALI conjunctival organotypic model. KRT7, KRT4 and MUC5AC were predominantly located in the superficial layer, while MUC4 was detected throughout (representative of repeat experiments in three different donor conjunctival ALI cultures). Hoe- Hoescht. Scale bars 50 µm.**B**) UMAP visualisation of scRNA-Seq data from conjunctival ALI cultures (534 cells from one donor) showing the presence of basal (CjB), suprabasal (CjSB) and superficial (CjS) conjunctival epithelium. Expression of SARS-CoV-2 entry factors, *ACE2* and *TMPRSS2* is shown as superimposed single gene expression plots on the UMAP.

Figure S2. Transmission electron microscopy of ALI conjunctival organotypic culture at day 30. A) Numerous apical microvilli are present on the surface of epithelial cells (arrows) indicating cell polarisation. **B**) Fluffy ocular surface-like electron dense glycocalyx (arrows) on surface of microvilli. **C**) Clear tight junctions (arrow) between cells. A-C: representative of repeat experiments in 3 donors. Scale bars 500 nm.

Figure S3. SARS-CoV-2 infection of human nasal ALI organotypic cultures and the impact of NF-κB activation on proinflammatory gene expression following SARS-CoV-2 infection of the ALI conjunctival model. A, B) Quantitative RT-PCR expression of nucleocapsid (*N*) gene (normalised to the housekeeper *RNASEP*) and subgenomic *N* RNA (normalised to *GAPDH*) of human nasal ALI organotypic cultures, MOI=0.1. Data shown as mean ± SEM, n=3-4 donors, * $p < 0.05$ unpaired T-test. **C)** Quantitative RT-PCR expression of nucleocapsid (*N*) gene (normalised to the housekeeper *RNASEP*) at 24 hpi. Data shown as mean ± SEM, n=2 experimental repeats from one donor. The NF-κB inhibitor was diluted in DMSO, hence a DMSO control was included. **D**) Quantitative RT-PCR expression of *CXCL8* and *TNFα* at 24 hpi. Data shown as mean ± SEM, n=3 experimental repeats from one donor. * $p < 0.05$, ** $p < 0.01$, *** $p < 0.001$, **** $p < 0.0001$, one way ANOVA with Tukey's multiple comparisons. **E)** ***IFITM1, 2, 3*** **expression in mock and infected SARS-CoV-2 infected conjunctival ALI cultures up to 72 hpi**. Data shown as mean ± SEM, n=3 donors. Conj – conjunctiva.

Table S1. A full list of highly and differentially expressed genes between the clusters identified in the ALI conjunctival organotypic culture at day 30 and 75 of differentiation.

Table S2. A full list of highly and differentially expressed genes between the clusters identified in the SARS-CoV-2 infected and mock ALI conjunctival organotypic culture at day 30 of differentiation.

Table S3. A list of differentially expressed genes (p adjust< 0.05) between infected and mock condition in all cell types.

Table S4. List of significant regulators of gene expression in the infected cells (versus mock) in all conjunctival epithelial cell types.

Table S5. List of IPA enriched pathways in infected cells (versus mock) in all cell types.

Table S6. A list of differentially expressed genes (p adjust< 0.05) between bystander and mock condition in all cell types.

Table S7. List of significant regulators of gene expression in the bystander cells (versus mock) in all cell types.

Table S8. A list of differentially expressed genes (p adjust< 0.05) between superficial conjunctival and secretory and ciliated nasal epithelial cells in the infected, bystander and mock groups.

Table S9. List of IPA enriched pathways in superficial conjunctival (versus secretory and ciliated nasal epithelial) cells in the infected, bystander and mock groups.

Table S10. List of significant regulators of gene expression in the superficial conjunctival (versus secretory and ciliated nasal epithelial) cells in the infected, bystander and mock groups.

Table S11. A List of oligonucleotides used in the quantitative RT-PCR analyses, together with the list of antibodies used in this study.

Supplementary Methods

Quantitative RT-PCR

RNA was extracted using TRIzol™ reagent (ThermoFisher Scientific), according to the manufacturer's instructions, and cDNA was generated using GoScript Reverse Transcription System following the manufacturers protocol (Promega). Viral RNA was detected using the CDC 2019-Novel Coronavirus Real-Time RT-PCR Diagnostic Panel as per Centre for Disease and Control's optimised protocol (Integrated DNA Technologies) for use with Go-Taq 1-step RT-qPCR Master Mix (Promega). Gene expression profile and SARS-CoV-2 N subgenomic RNA expression were determined with a standard qPCR cycle (50°C for 2 minutes, 95°C for 10 minutes, then 40 cycles of 95°C for 15 seconds, 60°C for 1 minute) using Go-Taq qPCR Master Mix (Promega) on a QuantStudio™ 7 Flex Real Time PCR System (ThermoFisher Scientific). Primer sequences can be found in **Table S11**. Data was interpreted using the ΔCT method.

Statistical Analysis

Statistical analysis was done using GraphPad Prism (version 9.0.0 121). Data was analysed with Ordinary one-way ANOVA using Tukey's multiple comparisons test unless otherwise indicated. Graphs are presented as mean ± SEM and are in Log10 format. * $p < 0.05$ ** $p < 0.01$ *** $p < 0.001$ **** $p < 0.0001$ ns – not significant.

Transmission Electron Microscopy (TEM)

Infected and mock samples were fixed overnight at 4°C in 2% glutaraldehyde (TAAB Lab Equipment) with 0.1 M sodium cacodylate (pH 7.4). The samples were then secondary fixed in 1% osmium tetroxide (Agar Scientific). Dehydration of samples was achieved with graded acetone (25, 50, 75, 100%) for 30 minutes each. Samples were impregnated with resin at the same graded concentrations up to 75% for 60 minutes each. A final incubation with 100% resin (minimum of 3 changes) for 24 hours at 60°C concluded the embedding process. Sections were taken at 70nm using a MT-XL ultramicrotome and mounted on a pioloform-filmed copper grid. Samples were stained in 2% aqueous uranyl acetate and lead citrate (Leica) and imaged on a Hitachi HT7800 transmission electron microscope. Representative micrographs were captured using an EMSIS Xarosa CMOS Camera with Radius software (version 2.1, EMSIS, Germany).

Immunofluorescence analysis (IF)

Inserts were fixed in 4% PFA for 1 hour and washed three times in 1xPBS. Two protocols were used to prepare tissue for staining. For apical side staining only, ThinCerts^{TM} were divided into four pieces and mounted on SuperFrost Plus^{TM} slides before staining. For apical and basal staining ThinCerts^{TM} were halved and embedded in OCT (Cellpath) then frozen at -20°C. Blocks were sectioned at 10µm on a Leica CM1860 cryostat to expose both the apical and basal sides of the tissue. Cryosections were dried, washed three times in 1xPBS and blocking solution was applied (10% donkey serum, 0.3% Triton-X in PBS) for 1 hour at room temperature. Primary antibodies (**Table S12**) were applied overnight at 4°C. Slides were washed three times with 1xPBS and secondary antibodies (**Table S12**) were applied for 1 hour at room temperature. AlexaFluora 488 and 546 (ThermoFisher Scientific) were used at 2ug/ml. Hoescht 33342 was added for 10 minutes and slides were washed three times in 1xPBS before mounted with VectaShield (Vector Laboratories). To detect the presence of mucins, Alcian Blue with periodic acid and Schiff's solution (PAS) staining was performed using the Alcian Blue PAS Stain Kit (ab245876, Abcam) according to the manufacturers' instructions. Neutral mucins were distinguished by magenta colour. All slides were imaged with an Axioimager Z2 microscope using the Apotome 2 system. Images were taken as Z-stacks and presented as maximum intensity projections (MIPs). Image acquisition was captured through Zen software.

Western Blotting

Lysis buffer (150mM sodium chloride, 1.0% NP-40 or Triton X-100, 0.5% sodium deoxycholate, 0.1% SDS (sodium dodecyl sulfate), 50mM Tris, pH 8.0)) was applied directly to the apical surface of ALI cultures and cells were removed by gentle scrapping. Protein concentration was determined by BCA Assay (ThermoFisher Scientific). NuPAGE^{TM} reducing agent and LDS sample buffer (ThermoFisher Scientific) were added directly and lysates were heated at 70°C for 10 minutes. 15µg of protein were ran on a Novex^{TM} Bolt 4-12% bis-tris mini gel with NuPAGE^{TM} 1x MES SDS running buffer (ThermoFisher Scientific). 5µl of PageRuler^{TM} Plus Prestained Protein Ladder (ThermoFisher Scientific) was used as a size guide. Proteins were transferred onto PVDF iBLOT 2 Transfer Stacks according to manufacturer's instructions (ThermoFisher Scientific). Membranes were blocked in 5% non-fat milk with 0.1% tween in PBS (PBS-T). Primary antibody combinations were incubated overnight at 4°C. Membranes were washed in 1xPBS-T and membranes were incubated in secondary antibodies for 1 hour at room temperature. Membranes were washed again in 1xPBS-T and developed with SuperSignal West pico

PLUS chemiluminescent substrate (ThermoFisher Scientific) according to manufacturer's instructions and imaged on an Amersham Imager 600.

Single Cell (sc) RNA-Seq sample processing

Single cell RNA-Seq was performed on cells harvested 24 hours post infection. ALI cultures were washed apically and basally in 1xPBS and dissociated in 100µl 0.025% Trypsin-EDTA (ThermoFisher Scientific) for 15 minutes at 37°C. Trypsin-EDTA was neutralised, and cells were counted on a haemocytometer to get an optimum concentration of 1000 cells per microliter. Suspensions were centrifuged at 400g for 3 minutes and resuspended in 0.04%BSA/PBS solution. For scRNA-Seq cells were captured and libraries generated using the Chromium Single Cell 3' Library & Gel Bead Kit, version 3.1 (10x Genomics). scRNA-Seq libraries were sequenced to 50,000 reads per cell on an Illumina NovaSeq 6000.

scRNA-Seq analysis

The sequencing data was aligned to human reference genome (GRCh38) and the Sars_CoV_2 reference (Ensembl ASM985889v3) using CellRanger Version 3.0.1. The Seurat R library was used for the downstream analysis. The filtered_feature_bc_matrix were imported into R and QC filtering applied. The thresholds were applied to the data were a minimum counts per cell of 2000, minimum genes per cell of 500 and maximum percentage mitochondria of 20%. The DoubletFinder package (McGinnis et al., 2019) was used to find and remove doublets. We performed two integrated analyses of the data. Firstly, the mock samples were combined to study the clustering profiles without infection and secondly the mock and exposed cells were integrated together. Seurat (Butler et al., 2018) was used to normalise the data and the first 2000 highly variable genes, identified through vst selection were chosen for the clustering analysis. The gene expression values were then scaled and the number of counts, number of genes and percentage of mitochondria reads per cell were regressed. A PCA dimension reduction was applied using the selected highly variable genes. This was followed by Harmony (Korsunsky et al., 2019) batch correction where batch was set to sample ID. We then constructed a shared nearest-neighbour graph using the first 10 components of the harmony embeddings. Clusters within the graph using a range of resolutions from 0.2 – 2.2. We chose a resolution of 2.2 which over-clustered the data then applied the FindAllMarkers function and annotated the clusters based on the expression of marker genes as either basal, suprabasal and superficial conjunctival epithelium, or fibroblasts. The exposed cells were then classified into infected

and bystander and differential expression was performed with the following contrasts: infected vs mock; bystander vs mock; and infected vs bystander. ISG and NFKβ target gene scores were generated using the AddModuleScore, which calculates the average expression for each group. The ISG gene list was taken from a published IFN-treated nasal cell dataset (Ziegler et al., 2020), while the NF-KB target gene list was obtained from an online resource (BostonUniversity, 2021). UMAP dimension reduction was performed and the DimPlot and FeaturePlot functions were used to visualise the clusters and expression of genes in individual cells and the DotPlot function was used to visualise the expression of genes within cell types and conditions.

Table S11.

List of oligonucleotides used in the quantitative RT-PCR analyses.

Oligos	Sequence
ACE2 Forward Primer	GAT CTT GGC TCA CAG GGG AC
ACE2 Reverse Primer	TGG CCT GTT CCT CAA TGG TG
TMPRSS2 Forward Primer	GAG GTG AAA GCG GGT GTG AG
TMPRSS2 Reverse Primer	TCA ACA GCA TCG AGT AAT GAT AGG T
KRT3 Forward Primer	TGC CGC AGA GTT CAT TCA GAC
KRT3 Reverse Primer	TGG TCT TGG GCA AAC GAC G
KRT6A Forward Primer	GTG TTG TGA ACC CCC ACC CA
KRT6A Reverse Primer	CGA AGA GCA CAG AAA TCA TCA CAG
KRT7 Forward Primer	ACA TCG AGA TCG CCA CCT AC
KRT7 Reverse Primer	ATA TTC ACG GCT CCC ACT CC
KRT12 Forward Primer	CCT CCA AAC CAT CAC CTT GGG
KRT12 Reverse Primer	AGC AGA ATC GGA AGG ACG CTG A
KRT14 Forward Primer	GTT CTC CTC TGG ATC GCA GTC
KRT14 Reverse Primer	CCA TGA CCT TGG TGC GGA T
KRT15 Forward Primer	GAA GTT GAG GGG AGT GCC TT
KRT15 Reverse Primer	TGG AGC AAG ATG ACT CAC AGG
MUC1 Forward Primer	TCT CAC CTC CTC CAA TCA C
MUC1 Reverse Primer	GAA ATG GCA CAT CAC TCA C
MUC4 Forward Primer	CTT ACT CTG GCC AAC TCT GTA GTG
MUC4 Reverse Primer	GAG AAG TTG GGC TTG ACT GTC
MUC16 Forward Primer	GCC TCT ACC TTA ACG GTT ACA ATG AA
MUC16 Reverse Primer	GGT ACC CCA TGG CTG TTG TG
S100A9 Forward Primer	ACA CAA ATG CAG ACA AGC AGC
S100A9 Reverse Primer	CAC CCT CGT GCA TCT TCT CG
TP63 Forward Primer	CTG GAA AAC AAT GCC CAG AC
TP63 Reverse Primer	GGG TGA TGG AGA GAG AGC AT
SARS-CoV-2 N sgRNA Forward	CTCTTGTAGATCTGTTCTCTAAACGAAC
SARS-CoV-2 N sgRNA Reverse	GGTCCACCAAACGTAATGCG
GAPDH Forward Primer	TGC ACC ACC ACC TGC TTA GC
GAPDH Reverse Primer	GGC ATG GAC TGT GGT CAT GAG
N1 Primer/Probe	2019-nCoV CDC EUA Kit Integrated DNA Technologies (IDT)
N2 Primer/Probe	2019-nCoV CDC EUA Kit Integrated DNA Technologies (IDT)
RNase P Primer/Probe	2019-nCoV CDC EUA Kit Integrated DNA Technologies (IDT)

A list of antibodies used in this study.

Primary Antibodies (IF)

Antibody	Species	Dilution	Stock Concentration	Manufacturer	Catalogue No	Lot No
K3/K7 (Keratin 3)	Mouse	1:50	1mg/ml	Millipore	CBL218	3101107
DeltaNP63	Mouse	1:500	Unspecified	Abcam	ab735	GR3310716-1
MUC4	Mouse	1:200	0.38mg/ml	Abcam	ab60720	GR3282887-2
MUC5ac	Mouse	1:200				
SARS Anti-Spike	Rabbit	1:50	0.5mg/ml	Novus Bio	NB100-56578	AB092903 C-09
Anti-hACE2	Goat	1:200	0.2mg/ml	R&D Biosystems	AF933	HOK0620051
TMPRSS2	Rabbit	1:1000	0.487mg/ml	Abcam	ab92323	GR3344246-1
LYPD2	Rabbit	1:200	Unspecified	Biorbyt	orb33258	CB13616
Cytokeratin 4	Rabbit	1:100	0.188mg/ml	Abcam	ab1599	GR149743
Cytokeratin 7	Rabbit	1:100	1.303mg/ml	Abcam	ab181598	GR3214132-15
Cytokeratin 12	Rabbit	1:50		Abcam	ab185627	
Cytokeratin 13	Rabbit	1:100	0.224mg/ml	Abcam	ab92551	GR211864-17

Secondary Antibodies (IF)

Antibody	Dilution	Stock Concentration	Manufacturer	Catalogue No	Lot No
Donkey Anti-Mouse	1:1000	2mg/ml	Thermo Scientific	A11055	1627966
Donkey Anti-Rabbit 546	1:1000	2mg/ml	Thermo Scientific	A10040	1504518

Primary Antibodies (Western Blotting)

Antibody	Species	Dilution	Stock Concentration	Manufacturer	Catalogue No	Lot No
SARS Anti-Spike	Rabbit	1:50	0.5mg/ml	Novus Bio	NB100-56578	AB092903 C-09
RSAD2	Rabbit	1:1000	Not available	Cell Signalling	13996	Not available
ISG15	Rabbit	1:1000	Not available	Cell Signalling	2743	Not available
GAPDH	Mouse	1:500	0.2mg/ml	Santa Cruz	SC-47724	I2418

Secondary Antibodies (Western Blotting)

Antibody	Dilution	Stock Concentration	Manufacturer	Catalogue No	Lot No
Swine Anti-Rabbit HRP	1:2000	0.26g/L	Dako	P0399	20069482
Rabbit Anti-Mouse HRP	1:2000	1.3g/L	Dako	P0260	20051347

References

BUTLER, A., HOFFMAN, P., SMIBERT, P., PAPALEXI, E. & SATIJA, R. 2018. Integrating single-cell transcriptomic data across different conditions, technologies, and species. *Nat Biotechnol,* 36**,** 411-420.

KORSUNSKY, I., MILLARD, N., FAN, J., SLOWIKOWSKI, K., ZHANG, F., WEI, K., BAGLAENKO, Y., BRENNER, M., LOH, P. R. & RAYCHAUDHURI, S. 2019. Fast, sensitive and accurate integration of single-cell data with Harmony. *Nat Methods,* 16**,** 1289-1296.

MCGINNIS, C. S., MURROW, L. M. & GARTNER, Z. J. 2019. DoubletFinder: Doublet Detection in Single-Cell RNA Sequencing Data Using Artificial Nearest Neighbors. *Cell Syst,* 8**,** 329-337 e4.

ZIEGLER, C. G. K., ALLON, S. J., NYQUIST, S. K., MBANO, I. M., MIAO, V. N., TZOUANAS, C. N., CAO, Y., YOUSIF, A. S., BALS, J., HAUSER, B. M., FELDMAN, J., MUUS, C., WADSWORTH, M. H., 2ND, KAZER, S. W., HUGHES, T. K., DORAN, B., GATTER, G. J., VUKOVIC, M., TALIAFERRO, F., MEAD, B. E., GUO, Z., WANG, J. P., GRAS, D., PLAISANT, M., ANSARI, M., ANGELIDIS, I., ADLER, H., SUCRE, J. M. S., TAYLOR, C. J., LIN, B., WAGHRAY, A., MITSIALIS, V., DWYER, D. F., BUCHHEIT, K. M., BOYCE, J. A., BARRETT, N. A., LAIDLAW, T. M., CARROLL, S. L., COLONNA, L., TKACHEV, V., PETERSON, C. W., YU, A., ZHENG, H. B., GIDEON, H. P., WINCHELL, C. G., LIN, P. L., BINGLE, C. D., SNAPPER, S. B., KROPSKI, J. A., THEIS, F. J., SCHILLER, H. B., ZARAGOSI, L. E., BARBRY, P., LESLIE, A., KIEM, H. P., FLYNN, J. L., FORTUNE, S. M., BERGER, B., FINBERG, R. W., KEAN, L. S., GARBER, M., SCHMIDT, A. G., LINGWOOD, D., SHALEK, A. K., ORDOVAS-MONTANES, J., LUNG-NETWORK@HUMANCELLATLAS.ORG, H. C. A. L. B. N. E. A. & NETWORK, H. C. A. L. B. 2020. SARS-CoV-2 Receptor ACE2 Is an Interferon-Stimulated Gene in Human Airway Epithelial Cells and Is Detected in Specific Cell Subsets across Tissues. *Cell,* 181**,** 1016-1035 e19.
